# Ureteral dilation recovery after intravesical reimplantation in children with primary obstructive megaureter

**DOI:** 10.3389/fped.2023.1164474

**Published:** 2023-06-23

**Authors:** Yan He, Xuemin Wu, Yingrui Xu, Zhaoquan Liu, Guoqiang Du, Xiangyu Wu, Wei Liu, Rongde Wu

**Affiliations:** ^1^ Department of Pediatric Surgery, Shandong Provincial Hospital, Shandong University, Jinan, China; ^2^ Department of Pediatric Surgery, Shandong Provincial Hospital Affiliated to Shandong First Medical University, Jinan, China

**Keywords:** primary obstructive megaureter, intravesical reimplantation, Cohen, pneumovesicum, ureteral dilation

## Abstract

**Background:**

To observe the postoperative recovery following ureteral dilation in primary obstructive megaureter (POM) after ureteral implantation, and evaluate the risk factors affecting ureter diameter resolution.

**Materials and Methods:**

A retrospective study was performed in patients with POM who underwent ureteral reimplantation using the Cohen procedure. Patient characteristics, perioperative parameters, and postoperative outcomes were also analysed. A widest ureteral diameter of <7 mm was defined as a normal shape and outcome. Survival time was defined as the time from surgery to ureteral dilation recovery or to the last follow-up.

**Results:**

A total of 49 patients (54 ureters) were included in the analysis. The survival time ranged from 1 to 53 months. The shapes of a total of 47 (87.04%) megaureters recovered, and most (29/47) resolutions happened within 6 months after surgery. In the univariate analysis, bilateral ureterovesical reimplantation (*p *= 0.015), ureteral terminal tapering (*p *= 0.019), weight (*p *= 0.036), and age (*p *= 0.015) were associated with the recovery time of ureteral dilation. A delayed recovery of ureteral diameter was noted in bilateral reimplantation (HR = 0.336, *p *= 0.017) using multivariate Cox regression.

**Conclusions:**

Ureteral dilation in POM mostly returned to normal within six postoperative months. Moreover, bilateral ureterovesical reimplantation is a risk factor for delayed postoperative recovery of ureter dilation in POM.

## 1. Introduction

Primary obstructive megaureter (POM) mainly caused by vesicoureteral junction obstruction is a well-known disease in pediatric urology (1), with an incidence of approximately 1 in 1,500 and a male preponderance (2). For POM patients with worsening hydroureteronephrosis, frequent urinary tract infection, and decreasing kidney function, the primary therapy is surgical treatment in the form of ureteral reimplantation (3). Of the numerous published studies, the improvement in renal function and descent of the renal pelvis anteroposterior diameter are used to assess the surgical outcome of hydronephrosis (4–6). However, to date, few studies have examined the changes in megaureter shape after ureteral reimplantation. In our previous study, the postoperative recovery of ureteral dilation in these patients differed among individuals. Hence, this study aimed to investigate the recovery of ureteral dilation for POM after surgery and the predictors that affect the resolution of ureteral dilation.

## 2. Methods

### 2.1. Patients

We retrospectively analyzed the medical history of patients with POM who underwent ureteral reimplantation using the Cohen method at our institution between January 2011 and January 2021. Pneumovesical Cohen has been the main approach used for POM in the last 5 years. POM was confirmed by diuretic provocation with 99mTechnetium mercaptoacetyltriglycine-3 (MAG3). Preoperative evaluations were performed using ultrasonography, computed tomography urography (CTU), magnetic resonance urography (MRU) and voiding cystourethrography (VCUG). After excluding the vesicoureteral reflux (VUR) through VCUG, megaureters with an obstructive pattern on diuretic renogram and the beak-like appearance or spindle-shaped narrowing of the ureterovesical junction on MRU or CTU are identified as the POM. For cases included in the present study, intraoperative findings and postoperative pathological results confirmed the diagnosis of POM. Hydronephrosis and ureteral diameter were assessed using ultrasound measurements recorded pre-operatively and during the postoperative follow up. The ureteral diameter was measured as the largest transverse dimension of the distal ureter, as shown on ultrasonography. Patients with POM secondary to ureterocoele, posterior urethral valves, or neurogenic bladder or those with other urinary system abnormalities were not included in the study. Cases without complete follow-up data within 1 year after surgery were also excluded.

### 2.2. Surgical procedures

Surgical indications included obstruction, as evidenced by hydroureteronephrosis with either reduced split renal function (less than 40% on the affected side) or symptoms (pain, breakthrough febrile urinary tract infection). All patients underwent surgery using Cohen's cross-trigonal ureteral reimplantation by a paediatric urologist. The pneumovesical approach was used as described by Yeung et al. (7). During the surgical procedure, the distal narrow segment and grossly dilated proximal segment of the ureter were dissected, and ureteral folding was performed if the ureteral diameter was >15 mm.

### 2.3. Follow-up

All patients underwent repeat urinalysis and ultrasound to monitor for obstruction at 1, 2, 3, 6 and 12-months postoperatively and once a year thereafter. If persistent changes in the general urine test and ultrasound were identified, VCUG and diuretic renography were performed. In our study, a widest ureteral diameter <7 mm under ultrasound was defined as the normal shape (3, 8) and outcome event. Survival time was defined as the time from surgery to ureteral dilation recovery or the last follow-up if the ureteral diameter did not become normal.

### 2.4. Statistical analysis

Statistical analyses were performed using SPSS version 25. Numerical variables are described as medians and quartiles. We used univariate Cox regression analysis for numerical variables and the log-rank test in the Kaplan–Meier estimation for categorical variables. Variables with a significance level below 0.05 were included in a multivariate Cox regression analysis. In multivariate analysis, *p *< 0.05 was considered to be statistically significant.

## 3. Results

A total of 49 patients (37 males and 12 females) were included in the analysis. The median age was 21 months (1–42 months), and the median survival time was 6 months (1–53 months). 25 of the patients were diagnosed prenatally, 13 were symptomatically detected, and 11 were incidentally diagnosed. The majority (31/49) were left-sided, 13 were right-sided, and 5 were bilateral. Of the 54 ureters, forty were operated using a laparoscopic pneumovesical approach, and 14 were treated via open surgery. Eight ureters underwent terminal ureteral folding (Table 1).

**Table 1:** Log-rank test in Kaplan–Meier estimation for categorical variables M (*p*25, *p*75).

Variables	Survival time (months)	*χ*^2^ value	*p*-value
Gender			
Male (42)	5.00 (2.00, 26.00)	1.128	0.288
Female (12)	6.00 (2.00, 11.00)		
Manifestations			
Prenatal diagnosis (28)	8.00 (2.00, 28.00)	2.074	0.355
Symptomatic (14)	4.00 (2.00, 16.00)		
Postnatal examination (12)	5.00 (1.00, 15.00)		
Side			
Left (36)	4.00 (2.00, 20.00)	0.800	0.371
Right (18)	11.00 (2.00, 28.00)		
Bilateral ureterovesical reimplantation			
No (44)	4.00 (2.00, 18.00)	5.949	0.015
Yes (10)	26.00 (15.00, -)		
Surgical procedure			
Pneumovesical (40)	6.00 (2.00, 26.00)	0.003	0.958
Open (14)	4.00 (3.00, 35.00)		
Ureteral terminal tapering			
No (46)	8.00 (2.00, 26.00)	5.521	0.019
Yes (8)	2.00 (2.00, 5.00)		

During the follow-up, three patients had febrile urinary tract infections within one month of the surgery and were treated with antibiotics, but they had no reflux according to a VCUG. One patient presented with mild urinary incontinence, and a VUR grade III was diagnosed using VCUG. Postoperative data, including parenchymal thickness (median 9.00 mm with IQR 7.00–12.00 mm vs. median 5.00 mm with IQR 3.00–6.75 mm), APD (median 6.50 mm with IQR 0.00–11.00 mm vs. median 16.00 mm with IQR 11.00–27.00 mm), widest ureteral diameter (median 3.00 mm with IQR 3.00–3.13 mm vs. median 15.00 mm with IQR 11.00–19.00 mm), were significantly improved (*p *< 0.001). No increase in ureteral dilation was observed during follow-up.

**Figure 1:**
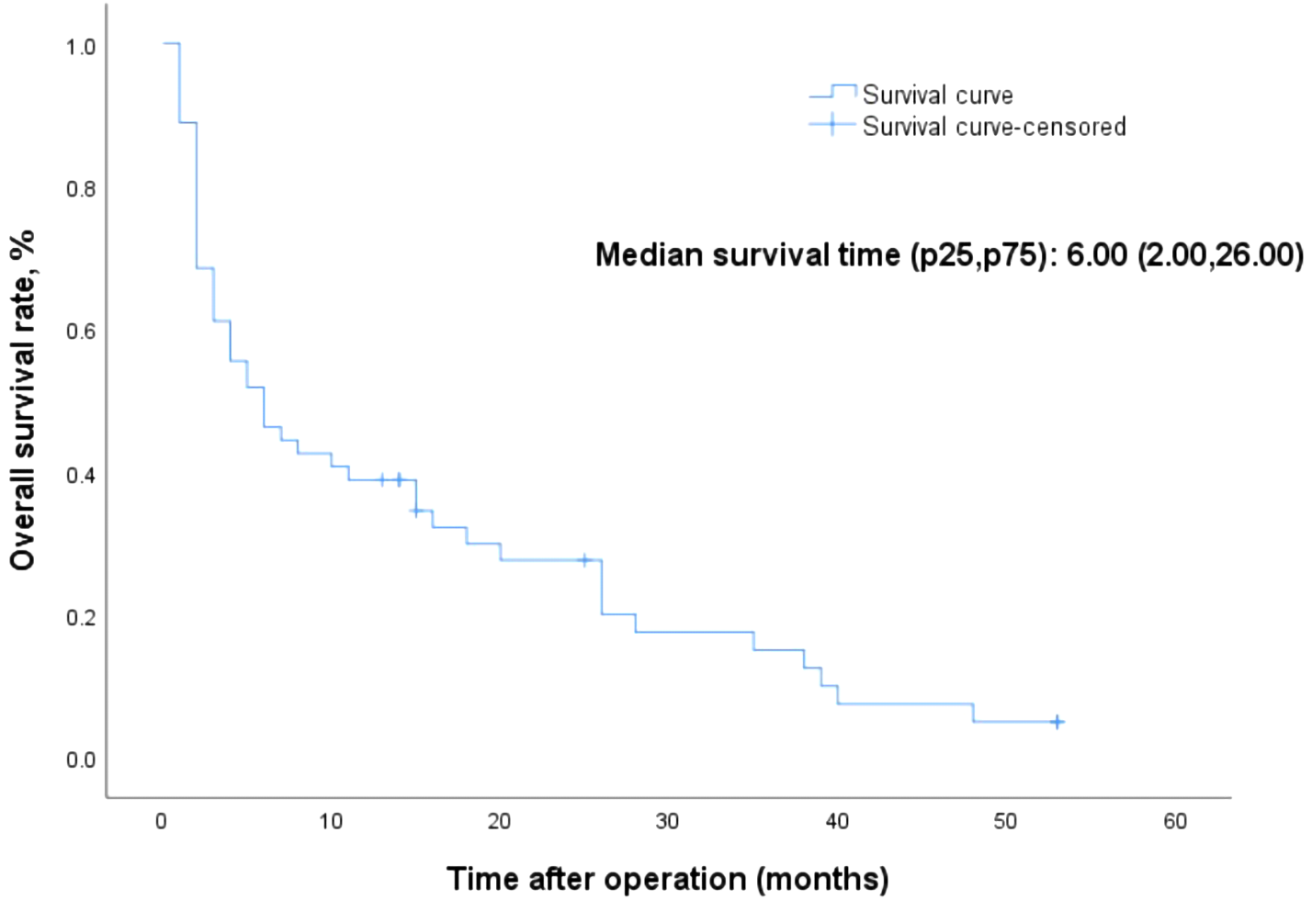
Kaplan-Meier survival curve for 54 ureters.

**Figure 2:**
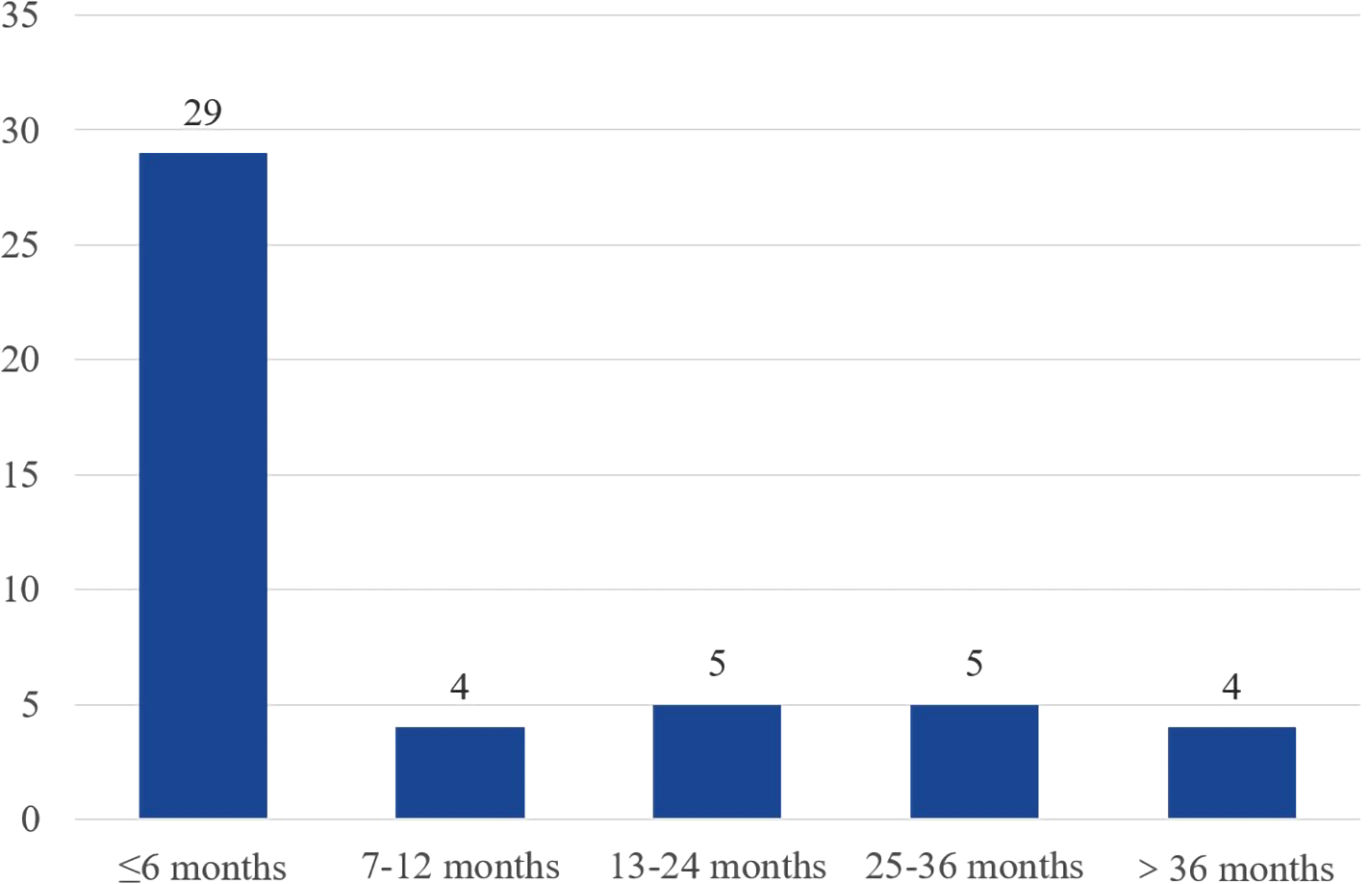
Distribution of ureter morphological resolution time in 47 ureters.

The Overall Kaplan-Meier survival curves are shown in Figure 1. The survival time ranged from 1 to 53 months, and the median survival time was 6 months. Forty-seven megaureter ureters progressively disappeared, and most resolution occurred within 6 months after surgery. Figure 2 shows the distribution of ureteral recovery time. There were seven censored events, and the median survival time for those cases was 15 months (range: 13–53 months). Except for two ureters without recovery to normal shape at the end of the study, five ureters were lost to follow-up. However, in those censored cases, the postoperative widest ureteral diameter was still reduced significantly (median 9.00 mm vs. median16.20 mm, *p *= 0.003).

**Figure 3:**
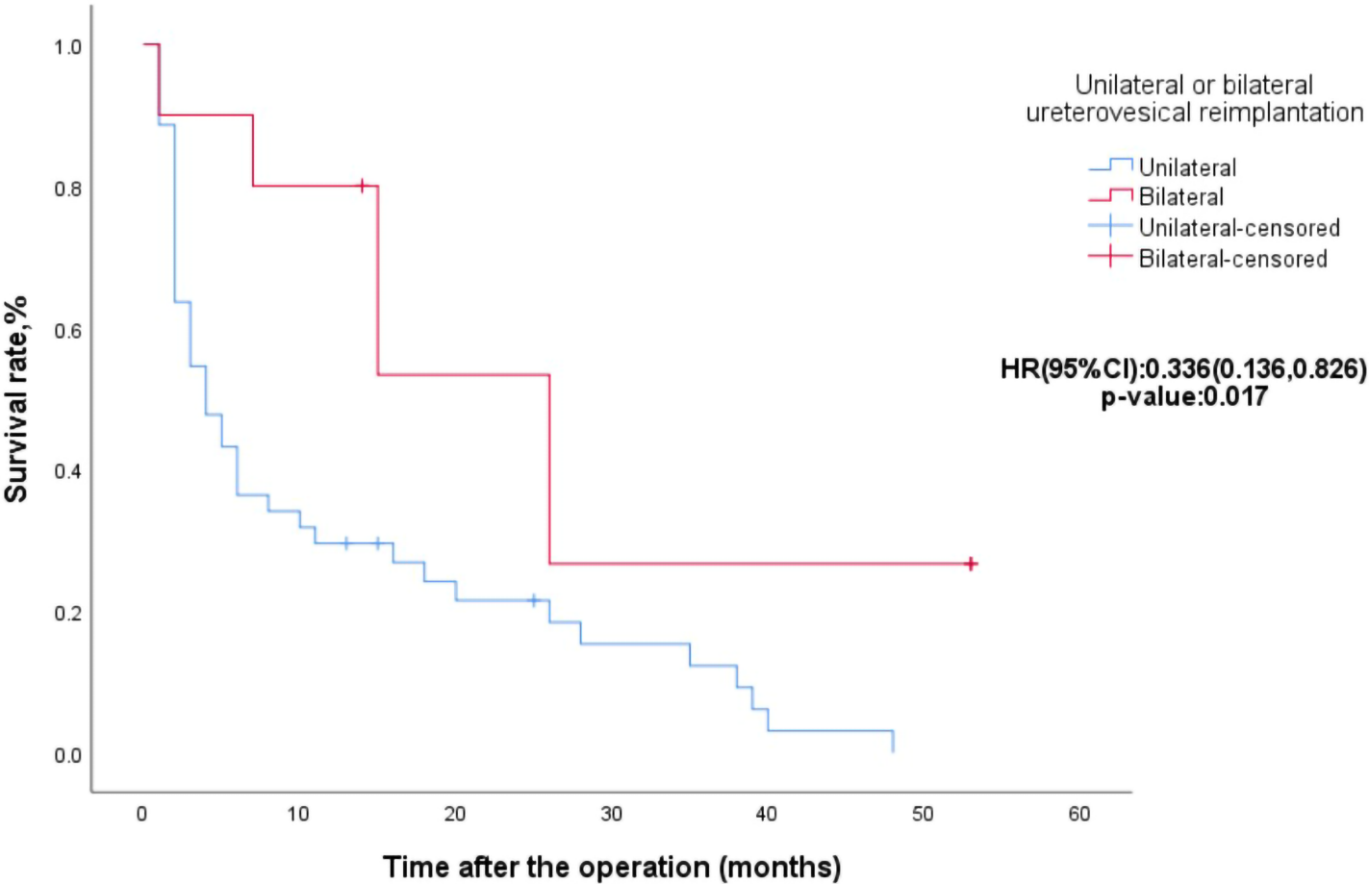
Kaplan-Meier survival curves for ureters with unilateral or bilateral ureterovesical reimplantation.

Tables 1, 2 show the univariate analysis results for clinical categorical variables and numerical variables related to the postoperative recovery time of ureteral dilation, respectively. Bilateral ureterovesical reimplantation (*χ*^2 ^= 5.949, *p *= 0.015), ureteral terminal tapering (*χ*^2 ^= 5.521, *p *= 0.019), weight (Wald = 4.417, *p *= 0.036), and age (Wald = 5.976, *p *= 0.015) were statistically significant. These factors were included in the multivariate analysis. Bilateral ureterovesical reimplantation (HR = 0.336, *p *= 0.017) was statistically significant in the multivariate Cox regression analysis (Table 3), and the Kaplan-Meier survival curves are shown in Figure 3. A slower decrease in dilatation of the megaureter to normal was noted in patients with bilateral ureterovesical reimplantation.

**Table 2:** Univariate Cox regression analysis for numerical variables (*n* = 54).

Variables	M(*p*25, *p*75)	Wald value	HR (95%CI)	*p*-value
Weight (Kg)	12.50 (10.00, 17.00)	4.417	1.049 (1.003, 1.097)	0.036
Age (months)	21.00 (11.75, 42.75)	5.976	1.016 (1.003, 1.028)	0.015
Parenchymal thickness (mm)	5.00 (3.00, 6.75)	0.709	1.056 (0.930, 1.199)	0.400
Postoperative anteroposterior diameter (mm)	16.00 (11.00, 27.00)	0.854	1.012 (0.987, 1.037)	0.355
Ureteral widest diameter (mm)	15.00 (11.00, 19.00)	0.587	1.020 (0.969, 1.074)	0.443
Ureteral terminal diameter^a^ (mm)	10.00 (6.00, 10.00)	1.217	1.089 (0.936, 1.267)	0.270
Submucosal tunnel length (mm)	30.00 (25.00, 35.00)	1.273	1.024 (0.983, 1.066)	0.259
Submucosal tunnel and ureter terminal diameter ratio	3.50 (3.00, 4.00)	1.052	0.817 (0.556, 1.202)	0.305

^a^ It means the diameter of terminal dilated ureter after tapering or not.

**Table 3:** Multivariable Cox regression analysis^a^.

Variables	*B* value	Wald value	HR (95%CI)	*p*-value
Bilateral ureterovesical reimplantation (No = 0, Yes = 1)	−1.092	5.646	0.336 (0.136, 0.826)	0.017
Ureteral terminal tapering (No = 0, Yes = 1)	0.681	2.012	1.975 (0.771, 5.060)	0.156
Weight (Kg)	0.045	0.686	1.047 (0.940, 1.165)	0.408
Age (months)	0.001	0.006	1.001 (0.970, 1.033)	0.939

^a^ According to the Omnibus test, −2log likelihood (−2LL) = 289.350, and the model was statistically significant (*p *= 0.007).

## 4. Discussion

Postoperative dilation recovery of the ureter and renal pelvis is a major concern in POM and hydronephrosis. Hydronephrosis after pyeloplasty has been reported to stabilise 6 to 8 months after the operation (9) and mostly resolves within 12 months (10). However, postoperative recovery of ureteral dilation in POM has rarely been reported. Cussen LJ has measured the ureteral shape of fetuses (>20 weeks) and children (<12 years), and then he concluded that the upper limit of normal ureteral diameters was 5.0–6.5 mm (11). In another study, after analyzing the radiological data of normal ureters from 194 children aged 0–16 years, Hellström M et al. defined the ureteral dilation as ureteral diameter >7 mm (12). On the basis of previous reports, in 2014, British Association of Paediatric Urologists confirmed that in fetuses after 30 weeks of gestation and children, a ureter can be thought of dilated if the diameter was larger than 7 mm (3). Furthermore, this criterion was also used in another study (8). In our study, ureteral diameter less than 7 mm was used as the cut-off value of the resolution of megaureter.

Ureterovesical reimplantation is currently the procedure of choice for POM (13). There have been many reports on the surgical procedure for ureterovesical reimplantation (14–16). Furthermore, in those articles, the degree of ureteral dilatation and hydronephrosis significantly improved after surgery. Similarly, we observed that the widest ureteral diameter recovered after surgery, and most megaureters (47/54) returned to a normal size. The resolution of the megaureter (29/47) mainly occurred within six months after surgery. What was striking was that we observed resolution even 36 months after the surgery. Therefore, long-term postoperative follow-up is necessary for children with persistent dilated ureters.

A multicentre international survey confirmed that bilaterality is a predictor of postoperative bladder dysfunction in extravesical ureteral reimplantation (17). In another study, bilateral primary obstructive megaureters appeared to have more postoperative vesicoureteral reflux after high-pressure balloon dilation (18). In our study, no postoperative bladder dysfunction or vesicoureteral reflux was observed in bilateral ureterovesical reimplantation. However, we found that ureters that underwent bilateral ureterovesical reimplantation had slower postoperative recovery of ureteral dilation. Therefore, leaving a stent in these cases would have contributed to ureteral recovery.

The role of ureteral tapering in the surgical repair of POM has been well documented and is controversial (19–21). Our clinical practice is to incorporate tapering when the distal ureteral diameter is greater than 1.5 cm to create an adequate anti-refluxing tunnel. Since the folding technique was judged to be better at maintaining the blood supply (22), we were more inclined to perform ureteral folding rather than tailoring. In this study, there was no significant risk effect of folding on the recovery of ureteral dilation, and there was no stenosis or reﬂux of the folding ureters during follow-up.

The relationship between age and postoperative recovery of the renal pelvis in patients with hydronephrosis has been previously reported. González Ruiz Y found that reduction of the postoperative renal pelvis anteroposterior diameter was more common in patients younger than one year (23). However, another study reported that early pyeloplasty (< 3 months) did not seem to contribute to the recovery of postoperative renal pelvis in children with prenatally diagnosed ureteropelvic junction obstruction (24). We incorporated age and weight into our analysis, but there was no significant association between age or weight and postoperative recovery of ureteral dilation.

## 5. Conclusions

Postoperative ureteral diameter in POM patients mostly returned to normal within 6 months. The resolution of ureteral dilation was slower in children with bilateral ureterovesical reimplantation, which is a risk factor for increased postoperative recovery time of ureteral dilation. The drawbacks of this study are its small size and the retrospective nature without renal function analysis. Long-term and large sample studies are needed for further exploration of ureteral dilation recovery after intravesical reimplantation.

## Data Availability

The raw data supporting the conclusions of this article will be made available by the authors, without undue reservation.
